# *De novo* purine synthesis reprograms the macrophage inflammatory response and the immune response in sepsis

**DOI:** 10.21203/rs.3.rs-8887742/v1

**Published:** 2026-02-19

**Authors:** György Haskó, Luyu Liu, Zoltán H. Németh, Gebhard Wagener, Ugur Akcan, Muhammed Arif, Pál Pacher, Taha Kelestemur

**Affiliations:** 1 Department of Anesthesiology, Columbia University, New York, NY, USA; 2 Purine Pharmaceuticals, Inc., East Hanover, NJ; 3 Department of Surgery, Morristown Medical Center, Morristown, NJ, USA; 4 Department of Neurology, Columbia University, New York, NY, USA; 5 Laboratory of Cardiovascular Physiology and Tissue Injury, National Institute on Alcohol Abuse and Alcoholism, National Institutes of Health, Bethesda, MD, USA

## Abstract

Sepsis is characterized by profound immunometabolic dysregulation, yet the role of purine precursor synthesis in immune reprogramming remains poorly defined. Intracellular purine nucleotides, such as ATP, are generated by *de novo* synthesis, which assembles purinosomes to build inosine monophosphate (IMP) from small precursors, or by the salvage pathway, which recycles purine bases such as hypoxanthine. Here, we investigated how these pathways regulate macrophage activation and host responses in sepsis. Silencing the *de novo* purine enzyme glycinamide ribonucleotide transformylase (GART) in LPS-stimulated macrophages induced marked transcriptomic remodeling, suppressing anti-inflammatory mediators, including IL-10 and TIMP-1, while increasing TNF-α. These effects were reversed by hypoxanthine supplementation, indicating rescue through salvage. Similar findings were observed with silencing of phosphoribosyl pyrophosphate amidotransferase (PPAT) or pharmacological GART inhibition with azaserine or lometrexol, which also reduced intracellular ATP levels in a hypoxanthine-reversible manner. In contrast, inhibition of salvage enzymes (HPRT, APRT) did not alter IL-10 expression. De novo purine synthesis blockade increased Adora2a expression and decreased Adora3 expression without affecting MAPK signaling. Macrophages formed purinosomes under purine-depleted conditions, which disassembled in the presence of exogenous hypoxanthine. In vivo, azaserine treatment in cecal ligation and puncture–induced sepsis reduced IL-10, increased TNF-α, and elevated bacterial burden. LPS-treated macrophages and PBMCs from septic patients showed reduced GART and PPAT expression. These findings identify *de novo* purine synthesis as a metabolic checkpoint that sustains anti-inflammatory macrophage programming and host defense, highlighting purine metabolism as a potential translational target in sepsis.

## Introduction

Sepsis is characterized by profound, system-wide metabolic dysregulation affecting both immune cells and parenchymal tissues. Innate immune cells undergo marked metabolic reprogramming, shifting from oxidative phosphorylation toward aerobic glycolysis, a pattern that supports rapid effector functions but promotes lactate accumulation, mitochondrial stress, and redox imbalance (1). During sepsis, intracellular ATP levels commonly decline in both immune and parenchymal cells as mitochondrial oxidative phosphorylation becomes impaired by inflammatory signaling, nitric oxide–mediated inhibition of the respiratory chain, and oxidative damage (2). Intracellular ATP is required for cell growth and biosynthesis, acting as the “energy currency” for metabolic activities. Glycolysis and oxidative phosphorylation in mitochondria are the primary biochemical pathways that produce ATP by phosphorylating ADP. While the process of intracellular ATP production from ADP has been widely studied, less focus has been placed on how ATP/ADP precursors like inosine monophosphate (IMP) and adenosine monophosphate (AMP) are synthesized in immune cells. Immune cells consistently recycle intracellular ATP even when they are in basal, resting, or unstimulated conditions (3). During periods of increased ATP demand, such as during immune stimulation and inflammation, including sepsis or cancer invasion, overall ATP turnover rises (4–7). Increased ATP turnover also occurs during hypoxia and ischemia, nutrient starvation, and glucose deprivation, all of which are part of the pathophysiology of sepsis (8–10). Enhanced ATP turnover may cause a net loss of precursor purines such as ADP, AMP, and IMP from cells unless they are replenished by increased synthesis. Intracellular IMP, which is the main precursor for AMP and subsequently ATP, is generated either by the *de novo* purine biosynthesis pathway or by recycling purine bases through the salvage pathway (11, 12).

De novo purine synthesis occurs in purine synthetic hubs, or “metabolons,” alternatively termed “purinosomes” (12–20). These are dynamic structures linked to mitochondria that contain enzymes essential for purine biosynthesis (21).

In the first committed step of de novo purine biosynthesis, 5-phosphoribosyl-1-pyrophosphate (PRPP) is converted to 5-phosphoribosylamine by glutamine-PRPP amidotransferase (PPAT). 5-Phosphoribosylamine is subsequently converted to inosine monophosphate (IMP) through a multistep pathway involving the sequential actions of the trifunctional enzyme GART, phosphoribosylformylglycinamidine synthase (PFAS or FGAMS), the bifunctional enzyme PAICS, adenylosuccinate lyase (ADSL), and the bifunctional enzyme ATIC (14).

PRPP also serves as the activated ribose donor in the purine salvage pathway, which recycles preformed purine bases—such as hypoxanthine, guanine, or adenine—derived from the diet or nucleotide turnover to regenerate purine nucleotides (14). This pathway is mediated by adenine phosphoribosyltransferase (APRT) and hypoxanthine-guanine phosphoribosyltransferase (HPRT)(22–24). APRT catalyzes the formation of AMP from adenine, whereas HPRT converts hypoxanthine to IMP and guanine to GMP (22–24).

Macrophages are central drivers of the inflammatory response during sepsis: they sense pathogens and tissue damage through pattern-recognition receptors, produce proinflammatory cytokines to recruit and activate other immune cells, and clear pathogens and debris at sites of injury (25). Importantly, they also orchestrate the resolution of inflammation by switching to anti-inflammatory and tissue-repair programs, including efferocytosis of apoptotic cells and secretion of pro-resolution mediators, thereby restoring homeostasis after injury (26). While the role of ATP generation from ADP through glycolysis versus oxidative phosphorylation in regulating macrophage function has been studied in detail (27, 28), the question of how purine generation through *de novo* synthesis and salvage regulates the macrophage inflammatory response has not been addressed in detail. In this manuscript, we investigate links between *de novo* purine synthesis and salvage and macrophage activation in both *in vitro* and *in vivo* models of sepsis.

## Materials and methods

### Ethics Statement

The studies involving human participants were reviewed and approved by the Columbia University Institutional Review Board (IRB) (Protocol # AAAS0172) and conducted under the Declaration of Helsinki principles. Suitable patients with sepsis admitted to the Surgical (SICU) or Cardiothoracic (CTICU) Intensive Care Units of Columbia University Irving Medical Center were identified by the study team and informed consent was obtained from the patient or their surrogates. We also obtained information from healthy control subjects prior to blood draws.

### Patient selection criteria

Selection criteria for septic patients were set up in accordance with The Third International Consensus Definitions for Sepsis and Septic Shock (Sepsis-3) (29). Sepsis was defined as an increase in Sequential Organ Failure Assessment (SOFA) score by 2 or more points (29).

### Isolation of peripheral blood mononuclear cells (PBMCs)

PBMCs were isolated using SepMate^™^ PBMC Isolation Tubes (STEMCELL Technologies, Cat. No. 86415) with Lymphoprep^™^ density gradient medium (STEMCELL Technologies, Cat. No. 07801), following the manufacturer’s instructions with minor optimizations. Venous blood was collected into 10 mL K_2_-EDTA-coated BD Vacutainer^®^ tubes (BD Biosciences, Cat. No. 366643). Whole blood was diluted 1:1 with phosphate-buffered saline (PBS) supplemented with 2% fetal bovine serum (FBS). The diluted blood was carefully layered along the walls of pre-prepared SepMate^™^ tubes containing Lymphoprep^™^, ensuring the total volume exceeded the insert level. Optimal separation was achieved with 5 mL of Lymphoprep^™^, though this volume was adjusted proportionally to the total blood–PBS volume when necessary. Samples were centrifuged at 1,200 × g for 10 minutes at room temperature with the brake enabled. After centrifugation, the plasma layer was collected from the top of the tube and stored at −80 °C for downstream applications, accounting for the twofold dilution introduced during sample preparation. The PBMC layer was collected either by swiftly pouring into a new 15 mL conical tube or by gentle aspiration with a 1 mL pipette. Cells were washed with PBS and pelleted by centrifugation. In cases of erythrocyte contamination, red blood cell lysis was performed with Hybri-Max^™^ Red Blood Cell Lysing Buffer (Sigma-Aldrich, Cat. No. R7757) for up to 5 minutes at room temperature. After lysis, cells were washed with PBS and pelleted. PBMCs were lysed with TRIzol^™^ (ThermoFisher Scientific, Waltham, MA, USA) and stored at −80 °C until further analysis.

### Mice

Experimentation on mice was approved by the Columbia University Institutional Animal Care and Use Committee (IACUC). Male C57BL/6 mice (10–12 weeks old) were purchased from Charles River Laboratories (Wilmington, MA). Animals were housed with up to five mice per cage in rooms with a 12-hour light–dark cycle under nonspecific pathogen–free conditions. All mice were housed for at least 1 week at the animal facility at Columbia University before experimental use and had access to regular chow and water *ad libitum*. Adult, age-matched mice were used for all experiments.

### Reagents and drugs

LPS (from *Escherichia coli*, serotype 055:B5), hypoxanthine, lometrexol-hydrate, bovine serum albumin (BSA), donkey serum, and thioglycolate medium were purchased from Millipore-Sigma (St Louis, MO, USA). DMEM, PBS, FBS, dialyzed FBS, and penicillin-streptomycin were obtained from Thermo Fisher Scientific. Azaserine was purchased from Medchemexpress (Monmouth Junction, NJ, USA).

### Cecal ligation and puncture (CLP)

Mice were injected intraperitoneally with either azaserine or vehicle (physiologic saline) 30 min before inducing sepsis. Polymicrobial sepsis was induced by subjecting mice to CLP (30–35). Mice were anesthetized with isoflurane (2%, 1 L/min) and the depth of anesthesia was checked by hind toe pinch reflex. Under aseptic conditions, a 2-cm midline laparotomy was performed to allow exposure of the cecum. Approximately two-thirds of the cecum was ligated with a 3–0 silk suture, and the ligated part of the cecum was perforated twice (through and through) with a 21-gauge needle (BD Biosciences, San Jose, CA, USA). The ligated cecum was gently squeezed to extrude a small amount of feces through the perforation site and was then returned to the peritoneal cavity, and the laparotomy was closed. After the operation, all mice were resuscitated with physiologic saline (1 ml injected subcutaneously) and returned to their cages, where they were provided free access to food and water. The mice were re-anesthetized with isoflurane 16–24 h after the CLP procedure, and blood and peritoneal lavage fluid were harvested.

### Preparation of peritoneal macrophages

Mice were injected intraperitoneally with 3 ml of 4% thioglycolate and peritoneal cells were harvested 3–4 days later. The cells were plated on 6- or 12-well cell culture plates at 200,000 cells/well and incubated in DMEM supplemented with normal (non-dialyzed) FBS and 100 mg/ml penicillin and streptomycin at 37 C in a humidified 5% CO_2_ incubator. Non-adherent cells were removed by rinsing the plates three times with cold PBS.

### Pharmacological treatment of macrophages for mRNA, cytokine, and intracellular ATP determination

Cells were treated overnight with various concentrations of azaserine or lometrexol, with or without hypoxanthine, in DMEM containing dialyzed FBS (purine-depleted). The next day, fresh DMEM with dialyzed FBS containing azaserine or lometrexol, with or without hypoxanthine, was added, immediately followed by 10 μg/ml LPS for 5 h for mRNA and ELISA assays, and for 6 h for intracellular ATP determination. At the end of the incubation, cells were collected in Trizol, supernatants were collected for cytokine measurements, or cells were lysed for intracellular ATP measurement.

### siRNA transfection and treatments

RAW 264.7 cells were plated 18 hours prior to transfection in 6- or 12-well plates. 750 μl of DMEM supplemented with normal (non-dialyzed) FBS (10% v/v) and 100 mg/ml penicillin and streptomycin was added to each well before transfection. Cells were transfected with 25–75 nM siRNA targeting GART (Catalog number #J-054838-09-0005, J-054838-10-0005, J-054838-11-0005, or J-054838-12-0005), PPAT (J-054694-06-0002, J-054694-05-0005, J-054694-07-0005, J-054694-08-0005), HPRT (J-045271-09-0005), or APRT (J-042945-12-0005), or with a control (D-001810-01-05) siRNA (all from Dharmacon) using GenMute^™^ Transfection reagent (SignaGen, Rockville, MD, USA). After 24 hours, the transfection medium was replaced with 1–2 ml DMEM supplemented with dialyzed FBS (10% v/v) and 100 mg/ml penicillin and streptomycin. After another 24 hours, the cells were treated with 10 μg/ml LPS for 30 min to 5 hours in medium containing dialyzed FBS 100 mg/ml penicillin and streptomycin. At the end of this incubation period, RNA or protein was collected, and the supernatants were harvested.

### Quantitative real-time PCR

Total RNA was extracted using TRIzol following the manufacturer’s protocol. Reverse transcription was then performed to synthesize complementary DNAs (cDNAs) using a High-Capacity cDNA Reverse Transcription Kit (Applied Biosystems, Waltham, MA). SYBR^®^ Green PCR Master Mix (Applied Biosystems) was used for quantitative analysis of gene expression levels. β-Actin or 18S served as an internal control, as indicated in the figure legends. Quantification of differences between groups was performed using the comparative threshold cycle (CT) method.

The murine primer sequences used were as follows: 18S forward: 5’-GTAACCCGTTGAACCCCATT-3’ and 18S reverse: 5’-CCATCCAATCGGTAGTAGCG-3’; TIMP-1 forward: 5’-TCCTCTTGTTGCTATCACTGATAGCTT-3’ and TIMP-1 reverse: 5’-CGCTGGTATAAGGTGGTCTCGTT-3’; β-actin forward: 5’-CGCCACCAGTTCGCCATGGA-3’ and β-actin reverse: 5’-TACAGCCCGGGGAGCATCGT-3’; IL-10 forward: 5’-AAGGGTTACTTGGGTTGCCA-3’ and IL-10 reverse: 5’-TTTCTGGGCCATGCTTCTCTG-3’; TNF-α forward: 5’-TCTTCTGTCTACTGAACTTCGG-3’ and TNF-α reverse: 5’-AAGATGATCTGAGTGTGAGGG-3’; ADORA2A forward: 5’-GAAGCAGATGGAGAGCCAAC-3’ and ADORA2A reverse: 5’-GAGAGGATGATGGCCAGGTA-3’; ADORA3 forward: 5’-TCATACCGGAAGGAATGAGC-3’ and ADORA3 reverse: 5’-AGCTTGACCACCCAGATGAC-3’; HPRT1 forward: 5’-ATACAGGCCAGACTTTGTTGGA-3’ and HPRT1 reverse: 5’-TGCGCTCATCTTAGGCTTTGTA-3’; GART forward: 5’-TCCTCAGGTCAAGCAAGTGTT-3’ and GART reverse: 5’-TGGTCCGACAACTACGAGTTC-3’; PPAT forward: 5’-TTCAGGGTGCATAAGGGAATGG-3’ and PPAT reverse: 5’-GCGTACCTCGTATGTCCGA-3’. For APRT, ATIC, PAICS, ADSL, and PFAS/FGAMS, PrimePCR assays were purchased from BIO-RAD.

For human HPRT, APRT, and 18S, PrimePCR assays were purchased from BIO-RAD.

### Western blotting

RAW 264.7 cells were collected and washed three times with prechilled PBS, then lysed in Radioimmunoprecipitation assay (RIPA) buffer supplemented with Phosphatase Inhibitor Cocktail (Sigma). The supernatant was collected after centrifugation at 12 000 g for 30 min at 4 °C, and the Pierce BCA assay (ThermoFisher) was used to determine the protein concentration. The cell lysate was supplemented with Laemmli SDS sample buffer (ThermoFisher), reducing (1X), and boiled for 5 min. Proteins were separated by SDS-PAGE and transferred onto 0.2 μm nitrocellulose membranes (Bio-Rad). The membranes were blocked with 5% (w/v) nonfat dry milk at room temperature for 1 hour to detect non-phosphorylated proteins, or with 5% (w/v) BSA to detect phosphorylated proteins. The membranes were incubated with the corresponding primary antibody overnight at 4 °C and washed with 1X Tris Buffered Saline with Tween-20 (TBST) three times. The membranes were then incubated with secondary antibodies at room temperature for 1 hour and washed with 1X TBST three times. The immunoblot bands were visualized using ChemiDoc Imaging Systems (Bio-Rad). The following antibodies were used: GART rabbit polyclonal antibody (Proteintech, 13659–1-AP, dilution 1:1000 in 1X TBST); PPAT rabbit polyclonal antibody (Proteintech, 15401–1-AP, dilution 1:1000 in 1X TBST); Stat1 rabbit antibody (Cell Signaling, Danvers, MA, USA, 9172T, dilution 1:1000 in 1X TBST); Phospho-Stat1 (Tyr701) (58D6) rabbit monoclonal antibody (Cell Signaling, 9167T, dilution 1:1000 in 1X TBST); p38 MAPK rabbit antibody (Cell Signaling, 9212S, dilution 1:1000 in 1X TBST); Phospho-p38 MAPK (Thr180/Tyr182) (28B10) mouse monoclonal antibody (Cell Signaling, 9216S, dilution 1:1000 in 1X TBST); SAPK/JNK rabbit antibody (Cell Signaling, 9252S, dilution 1:1000 in 1X TBST); Phospho-SAPK/JNK (Thr183/Tyr185) (G9) mouse monoclonal antibody (Cell Signaling, 9255S, dilution 1:1000 in 1X TBST); p44/42 MAPK (Erk1/2) (L34F12) mouse monoclonal antibody (Cell Signaling, 4696S, dilution 1:1000 in 1X TBST); Phospho-p44/42 MAPK (Erk1/2) (Thr202/Tyr204) rabbit antibody (Cell Signaling, 9101S, dilution 1:1000 in 1X TBST); HRP-conjugated GAPDH monoclonal antibody (Proteintech, HRP-60004, dilution 1:1000 in 1X TBST); b-actin mouse monoclonal antibody (Proteintech, 66009–1-Ig, dilution 1:1000 in 1X TBST).

### Cytokine assays

Cytokine concentrations in plasma and cell culture supernatants were determined using ELISA kits specific for murine cytokines. Levels of tumor necrosis factor (TNF-α), interleukin (IL)-10, and tissue inhibitor of metalloproteinases (TIMP)-1 were measured using ELISA duo sets purchased from R&D systems (Minneapolis, MN, USA). Plates were read at 450 nm using a Spectramax 190 microplate reader from Molecular Devices (Sunnyvale, CA, USA). Assays were performed according to the manufacturer’s instructions.

### RNA isolation and RNA-seq

Total RNA was extracted from fresh-frozen cell pellets using TRIzol and following the manufacturer’s instructions. The RNA samples were quantified using a Qubit 2.0 Fluorometer (ThermoFisher Scientific), and RNA integrity was assessed using the Agilent TapeStation (Agilent Technologies, Palo Alto, CA, USA). For library preparation, the NEBNext Ultra II RNA Library Prep Kit for Illumina and the NEBNext Poly(A) mRNA Magnetic Isolation Module (New England Biolabs, Ipswich, MA, USA), including clustering and sequencing reagents, were used according to the manufacturer’s recommendations. Briefly, mRNAs were enriched using Oligo(dT) beads. The enriched mRNAs were fragmented for 15 minutes at 94°C. First- and second-strand cDNA were synthesized. cDNA fragments were end-repaired and adenylated at the 3’ ends, and universal adapters were ligated to the cDNA fragments, followed by index addition and library enrichment by PCR with limited cycles. The sequencing library was validated on the TapeStation and quantified using a Qubit 4.0 Fluorometer (ThermoFisher Scientific) as well as by quantitative PCR (KAPA Biosystems, Wilmington, MA, USA).

The sequencing libraries were multiplexed and clustered onto a flow cell on the Illumina NovaSeq instrument according to the manufacturer’s instructions. The samples were sequenced using a 2x150bp paired-end (PE) configuration, targeting 20 million reads per sample. Image analysis and base calling were conducted by the NovaSeq Control Software (NCS). Raw sequence data (.bcl files) generated from the Illumina NovaSeq were converted into fastq files and demultiplexed using Illumina bcl2fastq 2.20 software. One mismatch was allowed for index sequence identification. After assessing the quality of the raw data, sequence reads were trimmed to remove possible adapter sequences and low-quality nucleotides using Trimmomatic v.0.36. The trimmed reads were mapped to the reference genome available on ENSEMBL using the STAR aligner v.2.5.2b. BAM files were generated as a result of this step. Unique gene hit counts were calculated using featureCounts from the Subread package v.1.5.2. Only unique reads that fell within exon regions were counted.

### Data Availability

The original data from this study will be made available upon reasonable request.

### Public Data Acquisition and Processing

Data were retrieved from a study using human PBMCs (36). Differential expression analysis was performed on data from the various septic and control group. The statistical analysis was performed using the DESeq2 pipeline in R, with the provided raw counts as input (37). A gene is considered significant if its False Discovery Rate (FDR) is below 5%. Normalized values (normalized log counts or TPM) were used to plot gene expression levels.

### Immunofluorescent detection of purinosomes and Adora2a in fixed cells

Immunofluorescent staining was performed to detect endogenous purinosomes in fixed cells using a standard protocol. 2 days before staining, RAW 264.7 cells were seeded onto 35 mm glass-bottom, tissue-culture–treated dishes at a density of approximately 5 × 10^4^ cells per dish in medium containing normal FBS. 1 day before staining, the medium on the cells was replaced with fresh medium containing normal or dialyzed FBS. After initial seeding and between medium changes, the cells were incubated at 37 °C in a humidified atmosphere containing 5% CO_2_ to allow proper attachment and growth. Cell adherence was confirmed by light microscopy, ensuring approximately 80% confluency. Culture medium was carefully aspirated on the day of staining, and cells were washed twice with warm PBS. Cells were then fixed by dropwise addition of 500 μL of 4% paraformaldehyde in PBS and incubated for 10 min at room temperature. After fixation, the paraformaldehyde solution was removed, and the cells were washed three times with PBS. For permeabilization, cells were incubated with 500 μL of 0.1% Triton X-100 (Sigma) in PBS, added dropwise, for 10 min at room temperature on an orbital shaker. The permeabilization solution was subsequently removed, and cells were washed three times with PBS. Non-specific binding was blocked by incubating the cells with 500 μL of blocking buffer containing 5% normal donkey serum in PBS with 0.1% Tween-20 (PBST) for 1 h at room temperature with gentle shaking. Following blocking, the buffer was aspirated, and cells were incubated with primary antibodies diluted in blocking buffer. For co-staining of PFAS/FGAMS and GART, rabbit polyclonal anti-PFAS antibody (A304–219A, Bethyl, Montgomery, TX) was used at a 1:500 dilution, and mouse monoclonal anti-GART antibody (H00002618-M01, Abnova, Taipei, Taiwan) was used at a 1:1000 dilution. Cells were incubated with the primary antibody solution overnight at 4 °C on an orbital shaker. The next day, the primary antibody solution was removed, and the cells were washed five times with PBST. Secondary antibody incubation was then performed using a 1:1000 dilution of CF488A-conjugated donkey anti-rabbit IgG and a 1:1000 dilution of CF568-conjugated donkey anti-mouse IgG, prepared in blocking buffer. Cells were incubated with the secondary antibody solution for 2 h at room temperature on an orbital shaker, protected from light to prevent photobleaching. After secondary antibody incubation, cells were washed four times with PBST, followed by a final wash with PBS alone to remove residual Tween-20. Cells were then maintained in PBS and imaged using a confocal laser scanning microscope with 20X objective (LSM 900, Zeiss, USA).

For Adora2a staining, RAW cells on 35 mm glass-bottom, tissue-culture–treated dishes were incubated in medium containing dialyzed FBS for 24 h. Thereafter, the medium was replaced with fresh medium containing dialyzed FBS, and the cells were treated with 5 mM azaserine or vehicle in the presence of LPS (10 mg/ml) for 5 h. The cells were then fixed and stained as described above. The primary antibody was obtained from Abcam (ab3461 at 1:20 dilution) and the secondary antibody was CF488A-conjugated donkey anti-rabbit IgG, as described above.

### Measurement of intracellular ATP levels

ATP was measured using a commercial ATP assay kit from Sigma (Cat # 119107).

### Statistical analysis

Values in the figures and text are expressed as mean ± standard error of means (SEM) of *n* observations. Statistical analysis comparing 2 groups was performed by 2-tailed unpaired student *t* test. For RNAseq data processing, after extraction of gene hit counts, the gene hit counts table was used for downstream differential expression analysis. Using DESeq2, a comparison of gene expression between the groups of samples was performed. The Wald test was used to generate P values and log2 fold changes. Genes with adjusted P values < 0.05 and absolute log2 fold changes >1 were called differentially expressed for each comparison. A PCA analysis was performed using the “plotPCA” function within the DESeq2 R package. The plot shows the samples in a 2D plane spanned by their first two principal components. GraphPad Prism 8.0.2 was used for data illustration. Results were considered statistically different when *p* was ≤ 0.05.

## Results

### Global changes in the transcriptome following GART silencing in LPS-stimulated macrophages

**Figure 1.**
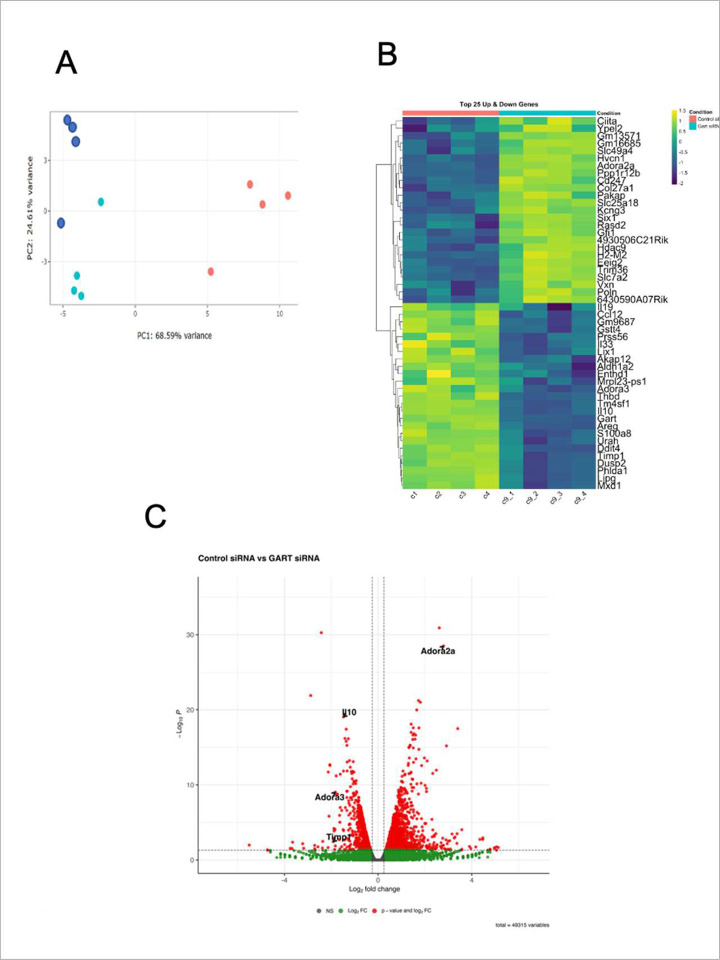
Inhibiting purine synthesis reprograms gene expression in LPS-stimulated RAW 264.7 macrophages. (A) RAW 264.7 cells were transfected with control or GART siRNA, and RNA-seq was performed after 5 h of LPS treatment in medium containing dialyzed FBS, which is depleted of hypoxanthine and other purine bases. In another group, GART-silenced cells were supplemented with exogenous hypoxanthine. The principal component analysis (PCA) figure displays a two-dimensional scatterplot of the first two principal components of the RNA-seq data. The control (dark blue), GART-silenced (orange), and GART-silenced with exogenous hypoxanthine treatment (light blue) groups are represented by different colors. Each dot represents a biological replicate of an RNA-Seq sample, with one tissue culture well as the origin of the sample. (B) Heatmap visualizing the expression profiles of a selected genes across biological replicates for Control siRNA (c1–c4; salmon top bar) and GART siRNA (g1–g4; turquoise top bar) conditions. Rows represent individual genes, and columns represent samples. The color scale indicates standardized expression values (row z-score) for each gene; yellow denotes high expression (upregulation), and purple denotes low expression (downregulation) relative to the mean. Hierarchical clustering of genes based on expression similarity is shown by the dendrogram on the left. (C) The global transcriptional changes between the control and GART-silenced groups in the absence of hypoxanthine were visualized by a volcano plot. The x-axis represents the fold change (FC), and the y-axis represents the negative of the adjusted p-value (-log10 Padj). The horizontal dashed line indicates the statistical significance threshold (Padj≤0.05), and vertical dashed lines indicate the FC thresholds (±0.25). Red dots represent significantly differentially expressed genes (DEGs) that meet both p-value and FC criteria. Green dots indicate genes meeting only the FC criteria, and grey dots represent non-significant (NS) genes.

To study the role of intracellular purine metabolism in regulating inflammation, we first silenced GART in RAW 264.7 macrophages, and then stimulated the cells with LPS. 5 hours after LPS stimulation, RNA was isolated from the cells, and RNA-seq was performed on the isolated RNA. GART silencing was done in both hypoxanthine-containing (with dialyzed FBS and exogenous hypoxanthine) and hypoxanthine-depleted (containing dialyzed FBS) medium to investigate the role of the salvage pathway as well. To explore the overall transcriptome structure, we performed Principal Component Analysis (PCA) on the top 500 most variable genes (using rlog-transformed counts). The PCA plot revealed a clear separation between control and GART-silenced cells along the first principal component (PC1), which accounted for 68.9% of the total variance, indicating significant differential expression between these conditions (Figure 1A). In addition, hypoxanthine treatment of GART-silenced cells largely but not completely reversed this separation, as control and hypoxanthine-treated GART-silenced cells clustered closely together (Figure 1A). Replicates within each group clustered tightly, confirming high reproducibility.

The heatmap of the top 25 up- and down-regulated genes confirmed that GART was efficiently silenced, as it was the among the most downregulated genes in the GART-silenced *vs*. control siRNA-treated cells (Figure 1B). The volcano plot showed a marked down-regulation of the major anti-inflammatory cytokine, *Il10* in the GART-silenced *vs*. control siRNA-treated cells (Figure 1C). Similarly, GART-silenced cells had downregulated expression of *Timp1*, another anti-inflammatory cytokine. In contrast, the expression of the proinflammatory cytokine *Tnf* was upregulated in GART-silenced *vs*. control siRNA-treated cells (not shown). Another notable molecule that was highly upregulated was the G protein-coupled receptor *Adora2a*, which we have shown to be an important modulator of macrophage function (38–40). In contrast, *Adora3* (41) was downregulated.

### GART and PPAT gene silencing suppresses LPS-induced IL-10 expression, an effect that is reversed by hypoxanthine

**Figure 2.**
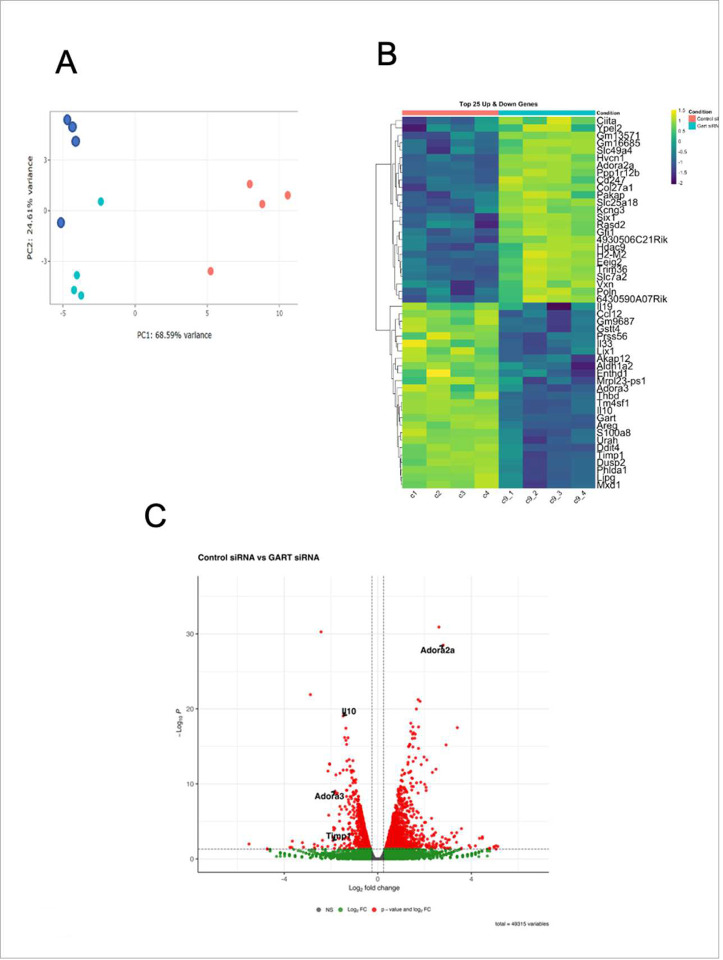
Inhibiting purine synthesis via gene silencing suppresses IL-10 gene expression in LPS-stimulated RAW 264.7 macrophages. GART silencing (A) in RAW cells decreases IL-10 mRNA (B) or secreted protein (C) levels, and this decrease is reversed by adding hypoxanthine (100 mM) to the otherwise hypoxanthine-depleted (containing dialyzed FBS) medium. PPAT silencing (D) in RAW cells decreases IL-10 mRNA (E) and protein levels (F), and this decrease is reversed by hypoxanthine supplementation. (G) GART or (H) PPAT silencing increases TNF-α mRNA, an effect reversed by hypoxanthine. In bar graphs, data are averages, and SEM, and each graph is representative of 2–3 experiments. *p≤0.05, **p≤0.01, ***p≤0.001.

Given that our RNA-seq demonstrated that *Il10* mRNA was down-regulated in GART-silenced *vs*. control cells (Figure 1C), and hypoxanthine treatment reversed this downregulation (data not shown), we studied the regulation of IL-10 in detail. Real-time PCR analysis confirmed that GART was efficiently silenced by the siRNA we used (Figure 2A) and that GART silencing suppressed IL-10 gene expression, an effect reversed by exogenous hypoxanthine (Figure 2B). ELISA showed that IL-10 secreted into the supernatant was reduced by GART silencing, and this reduction was reversed by hypoxanthine treatment (Figure 2C).

Silencing PPAT (Figure 2D) replicated the impact of GART silencing by lowering IL-10 levels (Figure 2E and F), and this effect was counteracted by hypoxanthine (Figures 2E and F). In contrast to IL-10, silencing either GART or PPAT led to increased mRNA levels of the proinflammatory cytokine TNF-a, an effect that was reversed by hypoxanthine (Figure 2G and H). GART silencing also suppressed the mRNA and secreted protein levels of another anti-inflammatory cytokine, TIMP-1 (Supplementary Figure 1A-C).

Finally, we evaluated whether, in the presence of functional *de novo* synthesis, salvage inhibition could regulate IL-10 or Adora2a gene expression. Our data showed that silencing either of the salvage enzymes HPRT or APRT did not affect IL-10 or Adora2a gene expression (Supplementary Figure 2A-D).

### Pharmacological GART inhibition suppresses IL-10 expression, an effect reversed by hypoxanthine

**Figure 3.**
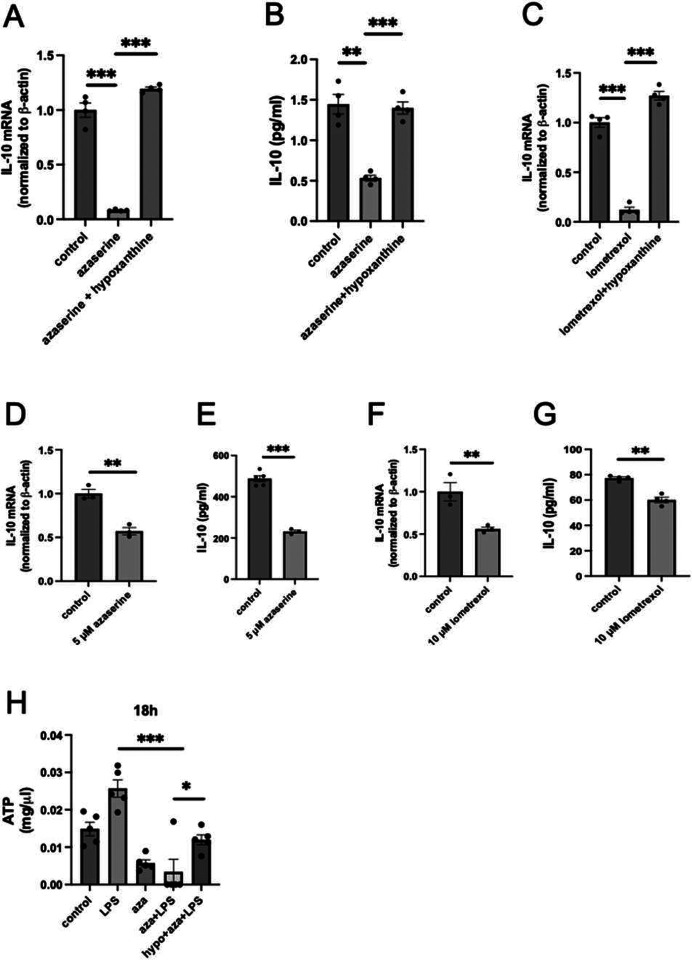
Pharmacological inhibition of *de novo* purine synthesis suppresses LPS-induced IL-10 production. Treating RAW 264.7 cells with azaserine (5 μM) reduces IL-10 mRNA (A) and protein (B) levels, and this effect is counteracted by adding hypoxanthine. (C) Lometrexol treatment in RAW cells reduces IL-10 mRNA levels, but this effect is reversed by adding exogenous hypoxanthine. Treatment of peritoneal macrophages with azaserine reduces IL-10 mRNA (D) and secreted protein (E) levels. Treatment of peritoneal macrophages with lometrexol reduces IL-10 mRNA (F) and secreted protein (G) levels. Azaserine reduces intracellular ATP levels in hypoxanthine-depleted medium (containing dialyzed FBS), and this effect is counteracted by exogenous hypoxanthine. In bar graphs, data are presented as averages and SEM, and each graph represents 2–3 experiments. *p≤0.05, **p≤0.01, ***p≤0.001.

In LPS-activated RAW 264.7 cells, pharmacological inhibition of GART with azaserine (13) decreased IL-10 mRNA and secreted protein in the absence of hypoxanthine, and supplementation with hypoxanthine reversed the azaserine-mediated suppression of *IL10* mRNA (Figure 3A). Azaserine also suppressed IL-10 release in the supernatant of RAW 264.7 cells, an effect that was countered by hypoxanthine (Figure 3B). Lometrexol, another GART inhibitor (42), also reduced *IL10* mRNA, an effect that was reversed by hypoxanthine (Figure 3C). In addition, both azaserine and lometrexol suppressed *IL10* mRNA and protein levels in primary, peritoneal macrophages (Figure 3D–G). We showed that azaserine suppressed secreted TIMP-1 protein, an effect reversible by hypoxanthine (Supplementary Figure 1A-C).

Finally, we confirmed that pharmacological inhibition of GART suppressed purine (ATP) synthesis, and that this decrease was reversed with exogenous hypoxanthine (Figure 3H)

### Intracellular purine synthesis differentially regulates Adora2a and Adora3

**Figure 4.**
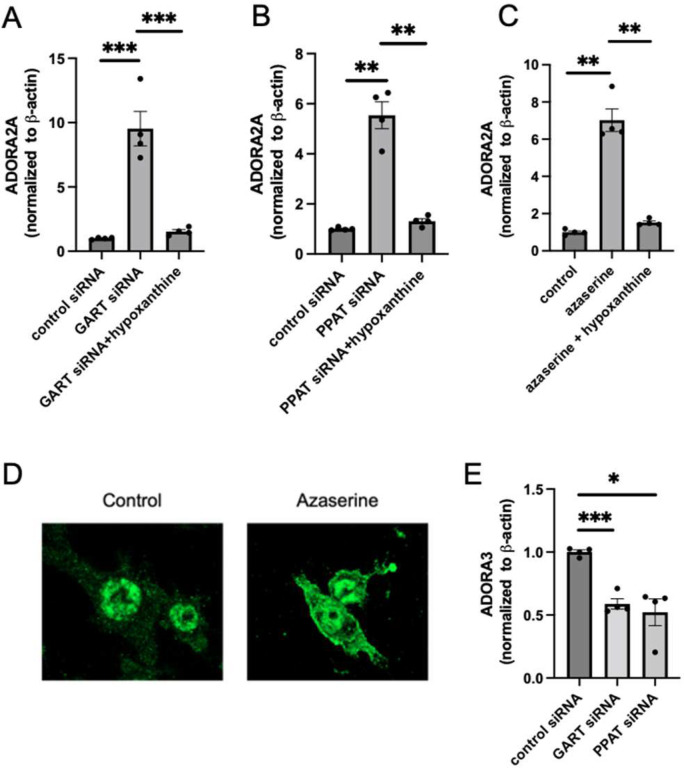
Inhibition of *de novo* purine synthesis augments Adora2a expression in LPS-stimulated RAW 264.7 macrophages. (A) GART silencing in RAW cells increases *Adora2a* mRNA levels, an effect reversed by hypoxanthine supplementation. (C) Silencing PPAT in RAW cells leads to an increase in *Adora2a* mRNA expression, and this effect is countered by supplementing with hypoxanthine. (D) Azaserine (5 mM) treatment of RAW cells increases *Adora2a* mRNA expression, an effect reversed by hypoxanthine supplementation. (E) Azaserine (5 mM) treatment of RAW cells shifts Adora2a protein expression from inside the cell to the cell membrane. Shown is 1 representative immunocytochemistry image from 3 experiments. Data were analyzed using confocal microscopy. (E) Both GART and PPAT silencing suppresses *Adora3* mRNA levels. In bar graphs, data are presented as averages and SEM, and each graph represents 2–3 experiments. *p≤0.05, **p≤0.01, ***p≤0.001.

Since *Adora2a* was among the most upregulated genes in cells in which GART was silenced (Figure 1C) and hypoxanthine reversed this upregulation (data not shown), we examined *Adora2a* regulation by intracellular purine metabolism in more detail. We confirmed through real-time PCR that silencing GART in LPS-activated RAW 264.7 cells increased *Adora2a* mRNA, and this effect was reversed by hypoxanthine (Figure 4A). Silencing PPAT also increased *Adora2a* mRNA levels in the absence of hypoxanthine, whereas hypoxanthine supplementation reversed the PPAT-mediated increase in *Adora2a* mRNA (Figure 4B). Azaserine also upregulated *Adora2a* mRNA, which was reversed by hypoxanthine (Figure 4C). Using immunofluorescence, we confirmed that azaserine caused Adora2a to relocate from inside the cell to the cell membrane (Figure 4D). In contrast to *Adora2a* mRNA, *Adora3* mRNA was suppressed in both GART- and PPAT-silenced cells (Figure 4E).

### Macrophages assemble purinosomes under purine-depleted conditions, and purinosomes disassemble in the presence of hypoxanthine

**Figure 5.**
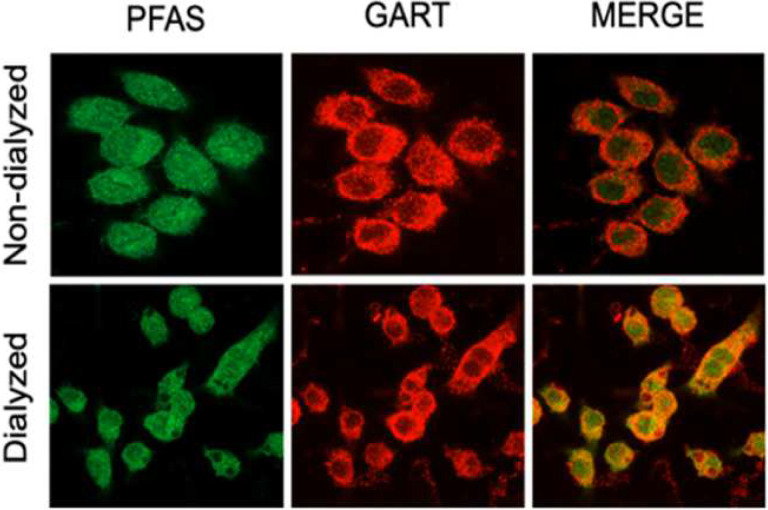
Colocalization of endogenous PFAS and GART for the visualization of purinosomes using immunofluorescence. Purine-depleted (by using dialyzed FBS) and repleted (by using non-dialyzed FBS) RAW 264.7 cells were fixed and permeabilized before being probed for PFAS with a rabbit polyclonal antibody and for GART with a mouse monoclonal antibody. Fluorescently labeled secondary antibodies, specifically CF488A-conjugated donkey anti-rabbit and CF568-conjugated donkey anti-mouse, were utilized to visualize the expression and localization of PFAS and GART, respectively. A representative image was captured using a 63× oil objective on a Zeiss LSM900 confocal laser scanning microscope. Sequential imaging of CF488A and CF568 revealed the colocalization of PFAS with GART, as indicated by the yellow puncta present in the merged image. Results from two experiments, each involving two different samples, are represented here.

The multiple enzymes involved in *de novo* purine synthesis assemble into purinosomes in live HeLa cells when extracellular purine levels (e.g., hypoxanthine) are low, and the purinosomes disintegrate in the presence of extracellular purines (12–14). This is because extracellular purines support the salvage pathway obviating the need for the assembly of enzymes of the *de novo* pathway and activation of *de novo* purine synthesis through the purinosome. While purinosomes have been observed in several cell types, they had not been detected in macrophages prior to our preliminary studies. The most widely used method to detect purinosome formation is to study colocalization of enzymatic members of the *de novo* purine synthesis pathway in the absence of extracellular purines. We investigated colocalization of GART and PFAS both in the presence or absence of hypoxanthine in RAW 264.7 macrophages, as colocalization of these two enzymes is widely used to detect purinosome formation (15). We demonstrated that in the absence of hypoxanthine, purinosomes formed based on confocal microscopic analysis of the colocalization of GART and PFAS (Figure 5 bottom). By contrast, upon inclusion of hypoxanthine in the medium, these two enzymes no longer colocalized indicating purinosome dissociation (Figure 5 top).

### GART silencing does not affect mitogen-activated protein kinase signaling in LPS-stimulated macrophages

**Figure 6.**
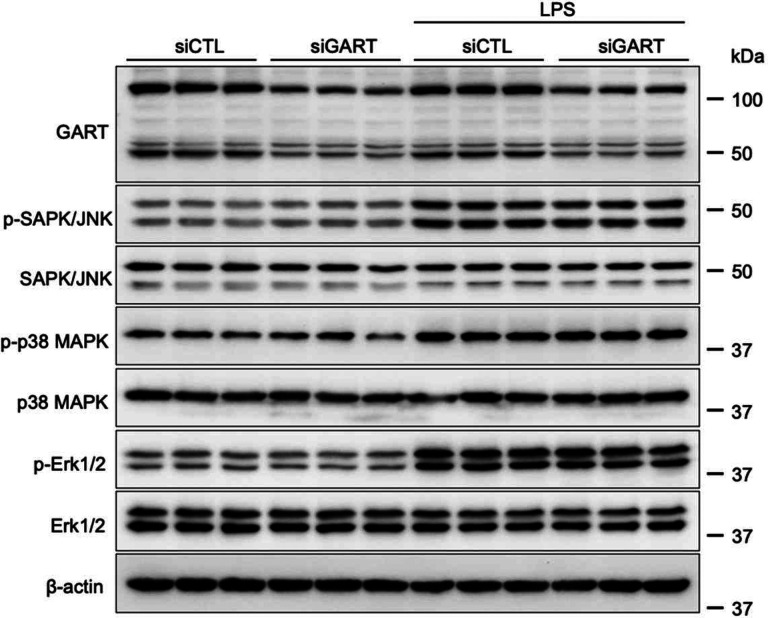
GART inhibition does not affect mitogen-activated protein kinase activation in LPS-stimulated RAW 264.7 macrophages. Control or GART-silenced macrophages were treated with LPS for 30 min. Activation of SAP/JNK, p38, and ERK1/2 was assessed in cell extracts by immunoblotting with antibodies specific for the active, doubly phosphorylated forms of these mitogen-activated protein kinases. Non-phosphorylated mitogen-activated protein kinases and β-Actin served as internal controls. Blots are representative of 3 independent experiments.

To further explore the connection between purine synthesis and the macrophage inflammatory response, we examined how GART-silencing impacts intracellular pathways typically linked to LPS stimulation. Our findings showed that silencing GART did not influence the LPS-induced phosphorylation/activation of the mitogen-activated protein kinases SAPK/JNK, ERK1/2, or p38 (Figure 6).

### LPS suppresses the expression of de novo purine synthesis enzymes in RAW 264.7 macrophages

**Figure 7.**
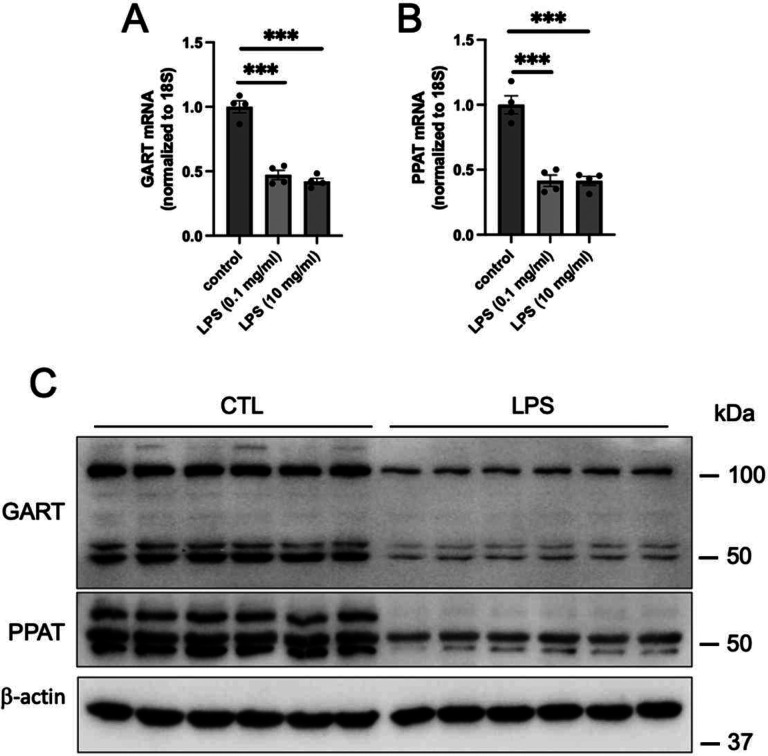
LPS suppresses GART and PPAT expression in RAW 264.7 macrophages. RAW 264.7 cells were treated with LPS for 5 hours at the indicated concentrations. *Gart* (A) and *Ppat* (B) mRNA levels were evaluated using real-time PCR. (C) RAW 264.7 cells were treated with LPS for 5 hours at 10 mg/ml. GART and PPAT protein expression was assessed by Western blotting, with β-Actin serving as the internal control.

We then studied how enzymes involved in *de novo* purine synthesis were regulated during inflammation and sepsis. We observed that a 5-hour LPS treatment of macrophages suppressed mRNA and protein levels of GART and PPAT (Figure 7A–C). In addition, LPS decreased mRNA levels of *Pfas*, *Paics*, *Adsl*, and *Atic* (Supplementary Figure 3A-D).

### Azaserine suppresses plasma IL-10 levels and increases TNF-α levels and bacterial numbers in murine sepsis

**Figure 8.**
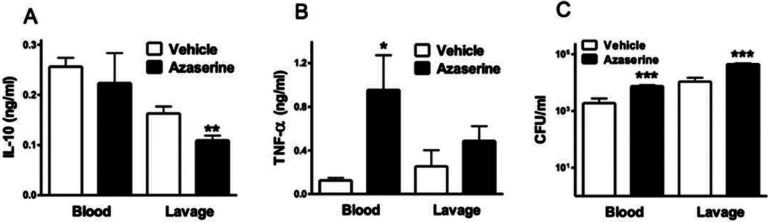
Azaserine differentially regulates IL-10 and TNF-α levels in sepsis. Mice received 20 mg/kg azaserine *via* intraperitoneal injection 30 minutes before sepsis was induced by CLP. Plasma and peritoneal lavage fluid levels of TNF-α (A) and IL-10 (B) were measured by ELISA. (C) Bacterial burden was assessed by counting colony-forming units (CFUs) on blood agar plates after serial dilution of blood and peritoneal lavage samples. Results are presented as mean ± SEM of n=8 in both groups (*p≤0.05; **p≤0.01; ***p≤0.005).

We then examined the role of purine synthesis *in vivo* during CLP-induced murine sepsis. Azaserine injection (20 mg/kg) 30 minutes before the CLP procedure reduced IL-10 levels (Figure 8A) and increased TNF-a levels (Figure 8B) in the peritoneal cavity and blood, respectively. These results align with our mRNA data from LPS-treated macrophages. Additionally, azaserine-treated mice showed an increased bacterial load (Figure 8C), indicating that purine synthesis is crucial for controlling bacterial dissemination.

### GART and PPAT levels decrease in PBMCs from human septic patients

**Figure 9.**
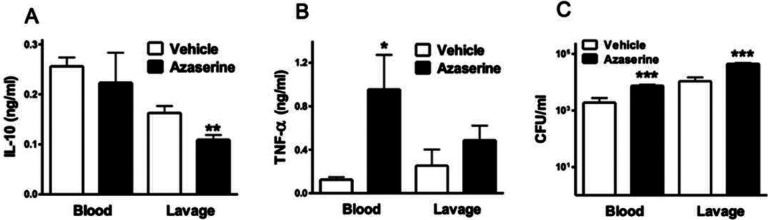
Sepsis reduces the expression of GART and PPAT in humans. mRNA expression of GART (A) and PPAT (B) in PBMCs of septic patients *vs*. healthy donors (controls). Every circle represents a different patient. PBMCs from septic patients and controls at New York Presbyterian Columbia were isolated as described in the methods section. CTL: control patients. In C and D, data were retrieved from published data on PBMCs in human patients as described in the results section. Differential expression analysis was performed of GART and PPAT from healthy controls (Hlty), patients with infection (Inf1), sepsis (Seps) or septic shock (Shock). Results are mean ± SEM (*p≤0.05; **p≤0.01; ****p≤0.0001).

mRNA levels of both GART (Figure 9A) and PPAT (Figure 9B) were lower in PBMCs but not neutrophils (data not shown) we isolated from septic patients at Columbia New York Presbyterian compared to healthy controls. We confirmed GART and PPAT downregulation using an RNA-seq database published in a prior work (36)(Figure 9C and D), where our analysis followed the methodology detailed in a manuscript we recently published (37). This indicates that the downregulation of GART and PPAT may also contribute to the decreased ATP levels observed during sepsis (43–45).

## Discussion

Sepsis has been shown to lead to a depletion of intracellular ATP levels in various tissues and cell types including PBMCs (43–45). During sepsis and shock, ATP is broken down into uric acid, resulting in a net loss of purines for the organism (46–48). ATP degradation occurs through adenosine, inosine, and hypoxanthine, which is the final purine that can be salvaged by the organism (49–52). Once hypoxanthine is converted to uric acid by xanthine oxidase, it is permanently lost from the body. Thus, how cells replenish intracellular purine levels and how purine synthesis is linked to inflammatory responses in immune cells are important questions.

Our study demonstrates that in macrophages, purine synthesis is linked to the inflammatory response in multiple ways. Overall, purine synthesis reprograms the macrophage transcriptome. More specifically, purine synthesis fuels the production of the major anti-inflammatory cytokine, IL-10. Moreover, purine synthesis is intimately linked to adenosine expression, where purine synthesis downregulates Adora2A and upregulates Adora3 expression. Exactly how purine synthesis leads to these alterations of the macrophage inflammatory response is unclear. For example, we find that activation of mitogen-activated protein kinases is not influenced by purine synthesis inhibition, indicating a role for other pathways. However, it is clear that both de novo purine synthesis and salvage can regulate IL-10 expression, as the decrease in IL-10 expression observed with blockade of de novo synthesis can be reversed by fueling salvage with exogenous hypoxanthine. Using HPRT1 knockout RAW 264.7 macrophages, a recent study demonstrated that purine salvage is required for optimal IL-10 induction; however, that study failed to find a role for *de novo* purine synthesis in regulating IL-10 production or in general macrophage function (53). This may be because they used LPS stimulation in conjunction with IFN-γ, whereas we stimulated cells only with LPS (53). Whereas LPS with IFN-γ completely shuts off *de novo* purine synthesis (53), we find that *de novo* purine synthetic enzymes are downregulated less following stimulation with only LPS. However, the less substantial downregulation of *de novo* enzymes observed with LPS alone in our study may allow these enzymes to function and contribute to purine synthesis and IL-10 induction.

Another question concerns the role of adenine-based purines, such as ATP, versus guanine-based purines, such as GTP, in mediating the promoting effect of purine synthesis on IL-10 gene expression and production. We show that blocking *de novo* purine synthesis decreases intracellular ATP levels, which are replenished by exogenous hypoxanthine, and that decreased IL-10 production is likewise reversed by hypoxanthine. However, our unpublished data (not shown) also indicate that IL-10 production can be reversed using guanine, suggesting that guanine-based purines may also play a role.

Our data demonstrating prominent regulation of Adora2a and Adora3 indicate that intracellular purine metabolism and extracellular signaling may interact. How this occurs remains to be determined, which is important given that adenosine receptors are powerful regulators of macrophage function, as we have demonstrated (38, 41, 54–67).

To our knowledge, we detected the purinosome in macrophages for the first time. This indicates that, in addition to regulating *de novo* purine enzyme expression, purinosome assembly and disassembly may represent another layer of regulation of purine production and inflammatory activation in macrophages. Clearly, a more detailed analysis of the regulation in macrophages is warranted.

Our data, based on the CLP model of sepsis and consistent with our *in vitro* findings, confirm that purine synthesis plays a crucial role in controlling the macrophage inflammatory response *in vivo*. An additional noteworthy point is that blocking purine synthesis not only affects cytokine regulation but also promotes bacterial dissemination, as evidenced by higher bacterial counts in the blood and peritoneal cavity of septic mice treated with azaserine. Therefore, the potential “energetic catastrophe” caused by reduced purine synthesis may also impair macrophages’ ability to kill bacteria. Moreover, the endogenous suppression of ATP production observed during sepsis (43–45) may precipitate bacterial spread.

Inhibiting xanthine oxidase with allopurinol and therefore preventing hypoxanthine breakdown reduces inflammation and improves metabolism post-sepsis, indicating a positive effect of the purine salvage pathway (68–70). Glutamine, an important precursor in the *de novo* purine synthesis pathway, offers an alternative to purine salvage by replenishing cellular ATP stores and enhancing metabolism and survival during sepsis (71, 72). Similarly, treatment of mice with AICAR, a central intermediate in *de novo* purine synthesis, ameliorated organ injury and reduced inflammation in septic mice (73–75). Therefore, restoring immune cell purine levels by fueling *de novo* purine synthesis or salvage could be a promising approach to treating human sepsis, especially given our data showing that *de novo* purine synthetic enzymes are downregulated in septic PBMCs.

## Supplementary Material

**Supplementary Figure 1. GART silencing suppresses TIMP-1 gene expression in LPS-stimulated RAW 264.7 macrophages.** GART silencing in RAW cells decreases TIMP-1 mRNA (A) or secreted protein (B) levels, and this decrease is reversed by adding hypoxanthine (100 mM) to the otherwise hypoxanthine-depleted medium. Treating RAW 264.7 cells with azaserine (5 μM) reduces TIMP-1 protein (C) levels, and this effect is counteracted by adding hypoxanthine. In bar graphs, data are presented as averages and SEM, and each graph represents 2–3 experiments. *p≤0.05, **p≤0.01, ***p≤0.001.

**Supplementary Figure 2.** Silencing of HPRT (A) or APRT (B) fails to affect *Il10* (C) or *Adora2a* (D) mRNA. In bar graphs, data are averages, and SEM, and each graph is representative of 2–3 experiments. *p≤0.05, **p≤0.01, ***p≤0.001. ns: not significant

**Supplementary Figure 3.** LPS suppresses *de novo* enzyme expression in RAW 264.7 macrophages. RAW 264.7 cells were treated with LPS for 5 hours at the indicated concentrations. *Pfas* (A), *Paics* (B), *Adsl* (C), and *Atic* (D) mRNA levels were evaluated using real-time PCR. In bar graphs, data are averages, and SEM, and each graph is representative of 2–3 experiments. *p≤0.05, **p≤0.01.

Supplementary Files

This is a list of supplementary files associated with this preprint. Click to download.

- Slide10.tiff
- Slide11.tiff
- Slide12.tiff
